# Neural correlates in the time course of inferences: costs and benefits for less-skilled readers at the university level

**DOI:** 10.3389/fpsyg.2026.1692021

**Published:** 2026-03-02

**Authors:** Mabel Urrutia, Esteban J. Pino, María Troncoso-Seguel, Claudio Bustos, Pamela Guevara, Karina Torres-Ocampo, Sandra Mariángel, Yang Fu, Hipólito Marrero

**Affiliations:** 1 Facultad de Educación, Universidad de Concepción, Concepción, Chile; 2 Facultad de Ingenieria, Universidad de Concepcion, Concepción, Chile; 3 Facultad de Ciencias Sociales, Universidad de Concepcion, Concepción, Chile; 4 Facultad de Humanidades y Arte, Universidad de Concepcion, Concepción, Chile; 5 Faculty of Education, Universidad Católica de la Santísima, Concepción, Chile; 6 Faculty of Communications and Arts, Universidad de las Américas, Concepción, Chile; 7 Institute of Language, Shanghai International Studies University, Shanghai, China; 8 Facultad de Psicologia y Logopedia, Universidad de La Laguna, San Cristóbal de La Laguna, Spain; 9 Instituto Universitario de Neurociencia (IUNE), Universidad de La Laguna, San Cristóbal de La Laguna, Spain

**Keywords:** event-related potentials, FN400, inferences, less-skilled readers, N400, post N400, slow negativity potential

## Abstract

This study investigates the costs and benefits of inference processing in university students with reading comprehension difficulties. Inferences, which combine implicit and explicit information, are seen as an indicator of better reading comprehension. The study focuses on three loci during narration: the last words of the first and second phrases, and a target word in a lexical decision task. Brain activity was recorded using event-related potentials (ERP) from 63 students as they read familiar, less-familiar, and neutral stories. The results revealed a slow negativity component at the first locus, with greater negativity for words from familiar contexts compared to less-familiar and neutral contexts. At the second locus, N400 and Post-N400 components reflected greater negativity for familiar contexts. At the third locus, FN400 and N400 components were observed for pseudowords. These findings suggest a bottom-up processing strategy, characterized by lexical access difficulties in less-skilled readers.

## 1 Introduction

Inferences are essential in the comprehension of a text as they integrate global knowledge into the language model (Prat and Yamasaki, 2015). They serve to complete the limited information in the text with semantic and pragmatic knowledge available in the memory of the individual in order to make sense of the reading (Bos et al., 2016; Cuetos et al., 2018). This study investigates a type of bridging inference, causal coherence inferences, whereby the representation of the general world, whether familiar or less-familiar, has an effect on reading comprehension (Noordman et al., 2015), in particular for university students with reading comprehension difficulties.

An important characteristic of inferences is their double semantic representation: textual representation and background knowledge that is activated in parallel during inference processing. However, there is a singularity: one of the sources of information has an advantage over the other and determines the time course of reading comprehension (Van den Broek et al., 2015). Thus, familiarity of information coming from the knowledge of the reader and their capacity to integrate it into the text through inferences will make a difference in university-level readers (Steele et al., 2013; Sundermeier et al., 2005). This dual processing of inferences implies the comprehension of implicit and explicit information (Van Overwalle and Vandekerckhove, 2013), whose level of activation varies according to reading skill. According to the results of the Programme for the International Assessment of Adult Competencies (PIAAC) (Arteaga, 2017), only 48.8% of young students worldwide make low-level inferences to comprehend texts. It is due to this that the study of this topic is of relevance to the field.

The main electrophysiological components that have been studied in inference generation are N400 and P600. The N400 component is a negativity that appears in the central parietal region between 300 and 500 ms when facing the probability of appearance of a stimulus (Kuperberg et al., 2020). On the other hand, the P600 component is a positivity that appears in the parietal region at around 500 ms when facing semantic review and repair processes; a re-analysis that is both syntactic and semantic due to an error in the interpretation of the phrase being read (Carretié and Iglesias, 1995). Some current studies have described another component called post-N400, which relates to the frontal-posterior regions and that relates to a response arising from a semantic violation surrounding a very specific context (Rodríguez Gómez, 2021). However, there is yet no clarity as to the behavior of such a component.

Electrophysiological measures have allowed for the determination of the time-course of cognitive processing in inference solving. Thus, the N400 effects have been reported in response to semantic anomalies that conflict with story’s broader context (Van Berkum, 2008). Additionally, the N400 component has been observed during the manipulation of socially incoherent inferences (Baetens et al., 2011; Leuthold et al., 2012). Tabullo et al. (2020) found an N400 component associated with unexpected endings coming from highly restrictive contexts during phrase reading.

On the other hand, the P600 component has been associated with context updating processes during inference processing (Baetens et al., 2011). Burkhardt (2007) also found a higher positivity, associated with the P600 components, in probable and inducible contexts in an experimental manipulation related to phrase reading. Kuperberg et al. (2020) found a P600 component in anomalous critical words that violated the restriction of the context, whether it was in low context or in a highly restrictive context, when compared to plausible critical words.

On the other hand, some experimental evidence with ERP shows that when the context is tightly related to the target word, the prediction of said word was high and, as a result, the amplitude of the N400 was lower (Baggio et al., 2008). Kuperberg et al. (2011) studied causal relationships in different scenarios. Their results show a higher amplitude of the N400 component in causally unrelated scenarios when compared to highly and intermediately causally related scenarios, regardless of whether appeared before or after the final position of the sentence. This shows the effect of contextual information on inference making. Ito et al. (2016) showed an attenuation of the N400 component for highly predictable as well as semantically related words when compared to unrelated words. Thus, the amplitude of N400 is modulated by the reader’s difficulty to integrate lexical information in a predictable context.

In spite of that, a type of N400 with a topographically left-frontal distribution has been discovered: the FN400 component, directly linked to familiarity in semantic language processing (Bridger et al., 2012). This is a neural correlate of familiarity that has been found in several recent investigations (Bader et al., 2023; Leynes and Upadhyay, 2022; Shayesteh et al., 2020; Stróżak et al., 2021). In Mecklinger and Bader’s (2020) meta-analysis, there is a discussion on a series of experiments that show this component as a quick evaluation of the fluency of ongoing processing, in relation to previous events or current expectations, which are related to the mechanism of relative familiarity. During an episodic recognition test, Leynes and Upadhyay (2022) found the FN400 component in pre-experiment familiar names, while pre-experimentally unfamiliar names evoked no old/new difference during the 300–500 ms interval. Bader et al. (2023) found the FN400 component in a recognition test where the effect of a previously named word or a new word was manipulated through a semantic judgment. The results support the notion that the FN400 takes place when fluency is attributed to familiarity during a recognition decision test.

One of the main inspirations of this research was that of Steele et al. (2013), who manipulated the familiarity variable in three contexts: familiar, less-familiar or neutral. After reading the phrases, a lexical decision task was done using words the participants may have inferred from the context or words that were unrelated to the scenarios. The results showed a reduction of the N400 component for words coming from unfamiliar contexts, as well as late positive activity. This was linked to the P600 for words coming from a familiar context when compared to a neutral context. The authors interpret such results as a marker of causal coherence inferences. In this research, we follow their experimental paradigm, with certain modifications to the SOA for the sake of contrast. Likewise, this research follows the time course of causal inferences starting from the context, through the critical phrase and up to the target, at three different moments of discourse comprehension.

Inferences that establish causal coherence are processed rapidly and automatically according to some authors (Hassin et al., 2002). The time course of such inferences is accelerated by context restrictions and, as a result, they are more likely to be generated (Griffiths and Tenenbaum, 2009). The manipulation of inferential context familiarity will evoke fewer alternatives for a representation of the event, therefore leading to a faster generation of inferences (Calvo, 2000). As the inferences in question are bridging or forward inferences—since the target word is related to the context of the story—, such inferences adjust better to McKoon and Ratcliff’s (1992; 1995) minimalist hypothesis. The authors’ theory posits that inferences are produced online only if they are supported by information coming from experience according to the situation model on general background knowledge.

The traditional position is that a less-skilled reader considers reading as a decoding task, while a skilled reader understands it as a meaning-building task (De Vega, 1984). Less-skilled readers have difficulties in particular while comprehending the vocabulary of difficult and meaningless words. Additionally, there are external consistency difficulties in the detection of information violating prior knowledge. On the other hand, they face difficulties in the detection of errors affecting the internal coherence of the text, especially in expository texts. In this regard, it is particularly difficult for less-skilled readers to access potential background information in the representation of discourse in order to build a coherent representation of the text during the course of reading (Ehrlich, 1996). In the case of Chilean university students, only 10% of them rank in the highest levels of reading comprehension (Centro de Estudios MINEDUC, 2016). The main difficulties they face are with implicit questions, such as linking information, making inferences, integrating information or transferring information coming from general knowledge to finally determine the global coherence of the text (OECD, 2019). Crucially, however, a significant gap remains in the literature: there are no prior studies investigating the time course of these inferential processes to reveal how online cognitive mechanisms unfold at three specific moments during the narration. This is particularly relevant as texts are processed incrementally, requiring readers to dynamically update their mental models as new information emerges (Pérez et al., 2024). Furthermore, there is a lack of evidence regarding how these real-time processes fluctuate across familiar and non-familiar texts, specifically within the population of university students struggling with reading comprehension.

The general aim of this research is to explore the costs and benefits of the time course of inference in university students with reading comprehension difficulties. The RI-Val model (O’Brien and Cook, 2016) provides a more mechanistic and dynamic account of inferential deficits in less-skilled readers compared to earlier minimalist perspectives. Accordingly, we hypothesize that struggling university readers will exhibit inefficient coordination among Resonance, Integration, and Validation processes. This dysregulation will be evidenced by specific electrophysiological patterns across three temporal stages of the narrative. Specifically, at the initial locus, familiar contexts are predicted to elicit a more pronounced N400 amplitude, stemming from a failure to inhibit competing textual data within the situation model. In later stages, we expect to observe N400 and post-N400 effects associated with sustained integration difficulties. These markers characterize the cognitive profile of less-skilled readers, whose processing remains constrained by local coherence at the expense of global narrative integration. Furthermore, given the participants’ deficits in Validation processes, lexicality (words vs. pseudowords) is expected to interact with context familiarity during inference generation, potentially eliciting FN400 or N400 effects as indicators of impaired conceptual mapping.

## 2 Materials and methods

### 2.1 Experiment design

In this study, a within-subject 3 context (familiar/less-familiar/neutral) by target lexicality (word/pseudoword) factorial design was defined. Three moments of the narration were recorded: locus 1, corresponding to the reading of the last word in the first phrase, which introduces the general context of the story; locus 2, corresponding to the reading of the last word in the second phrase, which is the critical phrase of the story; and locus 3, corresponding to the single target word derived from a lexical decision task. The independent variables were context [familiar (F), less-familiar (LF), and neutral (N)] and target lexicality (word or pseudoword presented as target). The dependent variable is the amplitude of the components in ERP responses and also the reaction times and accuracy rates for words and pseudowords in the behavioral lexical decision task corresponding to the target.

### 2.2 Participants

Sixty-three native Spanish speakers (56 women) ranging in age from 19 to 30 years (*M* = 21.3, SD = 2.31) volunteered for the experiment. The participants were undergraduate university students from the Faculty of Education who were pursuing a teaching program at a traditional university in Chile.

The students were enrolled in an elective course for improving reading comprehension which offered academic credits. Two diagnostic tests on reading comprehension revealed that 66% of the males and 68% of the females were below the reference score of the Inter-American Reading Test (Castañeda, 2015), which measures vocabulary knowledge test, ability to respond to a cloze test and response to questions about the meaning of a short text. Also, the students obtained a low percentage of reading comprehension and were ranked between the 78th and 79th percentile of the reference score of the Lectum test (Riffo Ocares et al., 2014), which measures long texts at a discursive level. Lectum is a diagnostic test used at the students’ university. When the scores were standardized to a 0 and 100 scale to indicate performance level, both the mean and the median are 50%. The minimum was 22%, and the maximum was 74%, far from the highest score. As a reference, in a similar population, 4 out of 6 men (66%) scored below 78 points, and 39 out of 58 women (68%) scored below 69 points, which corresponds to the cutoff score for the 50th percentile (Castañeda, 2015). Therefore, the participants of the current study have a performance level that is lower than expected for the university level.

To calculate the sample size, a repeated measures ANOVA test was considered, capable of differentiating by an intra-subject measure, context, which has three categories: familiar, less familiar, neutral. Assuming a large effect size (*f* = 0.5), with a power of 0.80 and a significance level of 0.05, a total of 40 participants were required. In order to address university dropout effect, which is common at this educational stage, we have considered a sample size almost a third more that which is required. From the original 63 participants, data from five participants were removed: one participant due to technical problems during experiment execution, four for having a low percentage of correctly recorded trials. Data from 58 participants were used for the analysis (51 women).

Additionally, the following exclusion criteria were considered: (a) a history of uncorrected sensory impairments (e.g., vision), (b) a history of neurological disorders (e.g., epilepsy, migraines, etc.), (c) a history of substance abuse, (d) a history of learning disorders (e.g., dyscalculia), (e) a history of language disorders (e.g., dyslexia, aphasia, etc.). All participants were right-handed with normal or corrected-to-normal vision. They received monetary compensation for transportation expenses. All participants received information on the experimental procedure and gave their informed consent before participating.

### 2.3 Stimuli

The material for the inference task was translated and adapted from the study of Sundermeier et al. (2005). The experiment consisted of 93 texts, each of which was made up by two sentences and a target word. A normative study was carried out to assess context familiarity of texts. Participants were asked to determine the degree of familiarity on a scale of 1–7, where 1 was LF and 7 was F. The results showed statistically significant differences between familiar and less familiar texts *t*(59) = 4.421, *p* = 0.0001. In addition, a normative study of cloze probability was conducted to determine the probability of the target word appearing in the experimental texts. Participants were asked to answer on a scale of 1–7 how likely it was that the target word would appear in the text, where 1 was very unlikely and 7 was highly likely. The results showed that there were no significant differences between the familiar and less-familiar variables. Therefore, in both contexts the target word could be inferred *t*(59) = 1.955, *p* = 0.057. All words and sentences endings were controlled for their length between F and LF sentences. Thus, in context sentence, there were no significant differences in length *t*(59) = −1.187, *p* = 0.240; in the critical sentence *t*(59) = 0.362, *p* = 0.240 and also in word target *t*(59) = 0 0.897, *p* = 0.374. In the same vein, the frequency of use of the words of the sentences was controlled according to Chilean lexical frequency (Sadowsky and Martínez-Gamboa, 2012): final word of context sentence *t*(59) = 0.951, *p* = 0.346; final word of critical sentence *t*(59) = 1.528, *p* = 0.132; target word, *t*(59) = 1.283, *p* = 0.204. Finally, we compared the imageability of the final words of familiar and less-familiar texts in the contextual sentence, *t*(31) = 1.447, *p* = 0.158, the critical sentence, *t*(31) = 0.510, *p* = 0.614; and the target words, *t*(31) = −1.690, *p* = 0.103. No significant differences were found in any of the comparisons.

### 2.4 Procedure and experimental paradigm

In the experimental session the appropriate size of the EEG cap was selected to fit each volunteer’s head. For the reading task, participants were seated in a height-adjustable chair, positioned in front of the stimulus presentation computer, which was a DELL computer, model G7 7790, featuring a 17.3-inch screen (1,920 × 1,080 pixels) and a refresh rate of 60 Hz, placed at 70 cm from the participant.

**FIGURE 1:**
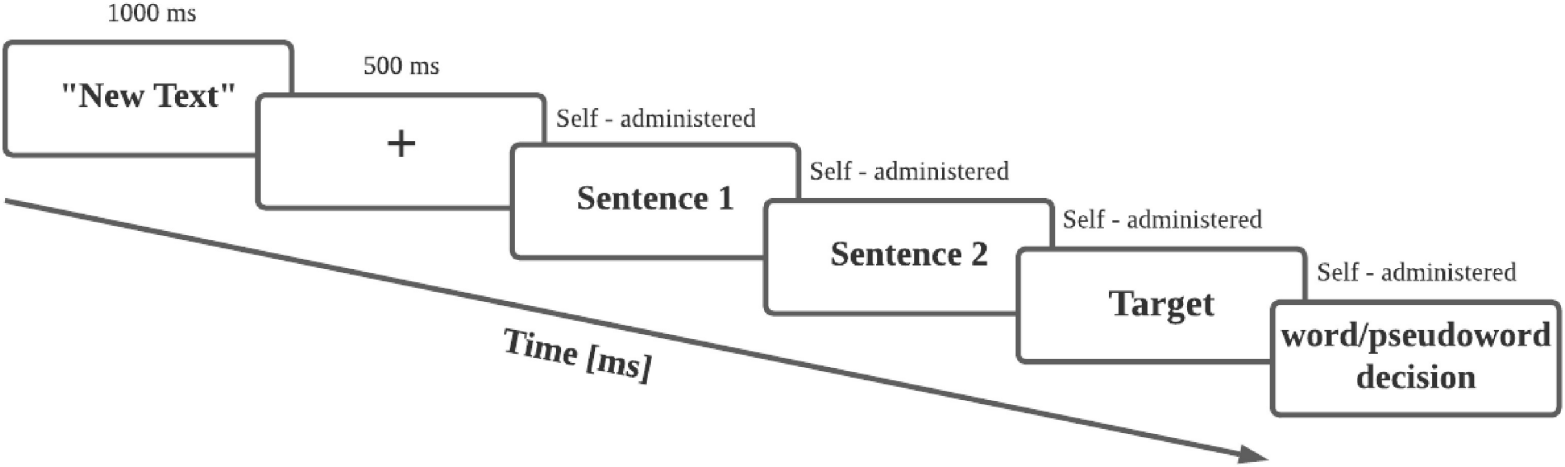
Experimental sequence diagram for each stimulus.

Experiment Builder software (SR Research Ltd, 2022) was used for stimulus presentation. First, the general instructions of the experiment were displayed on the screen. Then, a total of 93 stimuli were presented. For each stimulus, the context and critical phrase were presented as one-line text in the center of the screen, followed by a single target word. An example stimulus is shown in Figure 1. At the start, three stimuli served as practice trials. The experiment was divided into three blocks, each containing 30 stimuli, with breaks provided between each block to allow participants to rest. Stimuli were presented randomly within each block, with an equal distribution of F, LF, and N context (1/3 each). All experimental contexts were counterbalanced across participants. The target word was presented in the center of the screen, and participants were tasked with a lexical decision, determining whether the stimulus was a real word or a pseudoword through a YES/NO response. Half of the presented stimuli were words and half were pseudowords. In 30 out of the 93 stimuli, a comprehension verification question was included to assess text comprehension and ensure participants’ attention to the task (see Table 1). An external keypad was used to record participants’ responses. One key was used as “enter” to finish self-administered steps. A “yes” and a “no” keys were used to respond to the lexical decision (word/pseudoword) and to the attention questions.

**TABLE 1:** Example stimuli presentation.

Context	Sentence no.	Sentences	Presentation
Fixation point		+	1,000 ms
Familiar	1 (context)	Para preparar el examen, el estudiante abrió su libro. (*To study for the exam, the student opened his book).*	Self-administered (up to 3,500 ms)
	2 (critical)	Después de tres horas, estaba seguro de que conocía muy bien la materia. (*After three hours, he was sure that he knew the material well).*	Self-administered (up to 3,500 ms)
Less-familiar	1 (context)	El agente de la PDI recogió la fotografía del ladrón. (*The police officer picks up the thief’s photograph*).	Self-administered (up to 3,500 ms)
	2 (critical)	Después de unos minutos, se sintió seguro de que podría identificar al hombre en la calle. (*After a few minutes, he felt confident that he could identify the man on the street*)	Self-administered (up to 3,500 ms)
Neutral	1 (context)	Los niños llegaron a su primer día de escuela. (*The children arrived at their first day of school*)	Self-administered (up to 3,500 ms)
	2 (critical)	Estaban emocionados de conocer a su nuevo profesor, hacer nuevos amigos y jugar en el recreo. (*They were excited to meet their new teacher, make new friends, and play at recess*)	Self-administered (up to 3,500 ms)
Target word	Task	ESTUDIO (Study)	Self-administered Self-administered
Yes/No question		Is it a word? Yes/No answer through button box (word/pseudoword evaluation)	
Post sentence blank screen			1,500 ms

The experimental session lasted on average 40 min per participant.

### 2.5 EEG data recording

During the experiment, EEG data were collected using a sixty-four active electrodes actiCAP elastic cap by Brain Products (Brain Products GmbH, 2022a) (FP1, FP2, AF7, AF3, AFz, AF4, AF8, F7, F5, F3, F1, Fz, F2, F4, F6, F8, FT9, FT7, FC5, FC3, FC1, FC2, FC4, FC6, FT8, FT10, T7, C5, C3, C1, Cz, C2, C4, C6, T8, TP9, TP7, CP5, CP3, CP1, CPz, CP2, CP4, CP6, TP8, TP10, P7, P5, P3, P1, Pz, P2, P4, P6, P8, PO7, PO3, POz, PO4, PO8, O1, Oz, O2, Iz), positioned according to the international 10/10 system. The reference electrode is located in the central area of the cap (FCz), and the ground electrode on the forehead.

To set up the experiment, the electrodes were first connected to the actiCAP ControlBox to assess contact impedance. This set-up was performed for each participant, to ensure that all 64 channels’ impedance was lower than 25 KΩ before running the experiment.

The recording of the data for each participant was performed by connecting the electrodes to a standard BrainAmp amplifier, which was isolated from the power supply line by means of Brain Products Power Pack batteries. Each amplifier was connected, via fiber optic, to a USB-BUA128 adapter that transferred the signals in real time via USB port and cable to a laptop running BrainVision Recorder (Brain Products GmbH, 2022c) capture software. Data were recorded using a sampling rate of 500 Hz.

Synchronization signals were sent from Experiment Builder software to Brainvision recorder software to mark the display of the target on screen and at the participants’ response to the lexical decision.

### 2.6 EEG data pre-processing

EEG data were processed using Brainvision Analyzer (Brain Products GmbH, 2022b). The pre-processing pipeline included a visual inspection of EEG channels (Antúnez et al., 2022; Degno et al., 2021; Dimigen and Ehinger, 2021; Scharinger et al., 2020). In case of channel rejection, the channel was interpolated from neighboring electrodes. Then, all channels were re-referenced to the mean, and all channels were subsequently filtered from 0.1 to 30 Hz with an 8th order FIR filter, followed by a notch filter. Then, a visual inspection to detect artifacts using a semiautomatic method with a threshold of 150 μV and a gradient of 50 μV/ms was done. Also, ocular artifact correction using ICA from Brainvision Analyzer was used, with FP2 (vertical) and F8 (horizontal) references. EEG data were segmented into epochs of interest: −200 to 1,000 ms time-locked to the target word onset. The individual mean was calculated grouping by context (F, N, LF) and by lexicality (word, pseudoword), without considering the segments that were marked as faulty during visual inspection. Then, a baseline correction from −200 to 0 ms was applied. Finally, epochs were averaged to obtain the grand average for each stimulus, loci 1, 2, and 3. These were later grouped by context and by lexicality for visual exploration of the different components.

### 2.7 Data analysis

A quantitative analysis was conducted, including descriptive statistics (measures of central tendency and dispersion), as well as inferential statistics using a linear mixed-effects model on the averaged data with random intercept for subjects. Statistical power and effect size are reported for the results obtained.

All analyses were performed with R software with the “lme4” package for linear mixed-effects modeling (RStudio Team, 2022). For ERP analyses, a massive univariate analysis was performed to evaluate the effects of the 3 × 2 design. For each channel and each time point, cluster permutation was performed. This analysis was broken down by context, for locus 1 and 2, and additionally by lexicality in the case of locus 3.

For the analysis of locus (1 and 2) in mix model we only included the context as a fixed effect. However, for the analysis of locus 3 (target word), we included the interaction between context (F, LF, N) and lexicality factors (word, pseudoword). For random effects, an intercept-only model was used, which represents the subject effect.

We used the ANOVA output of lmer (Type III with Satterthwaite’s method considering a significance level of 5%) and R emmeans package to analyze contrasts. Additionally, *p*-values were adjusted using the Tukey method.

For reaction times in the behavioral analyses of the lexical decision task, the same mixed-effects model as for locus 3 was applied, with the difference that trial was included as an additional random effect. Additionally, we used the ANOVA output of lmer and explored contrasts. Similarly, for the analysis of task accuracy, by used a generalized mixed-effects model with binomial family considering correct and wrong answers in the model. For random effects, an intercept-only model was used, which represents the subject effect. We presented the summary and ANOVA output results of the model.

## 3 Results

In this section, we show the results of the analysis of each type of the studied locus: locus 1, locus 2 and locus 3 in three different segments of the text.

### 3.1 Locus 1: slow negativity potential

**FIGURE 2:**
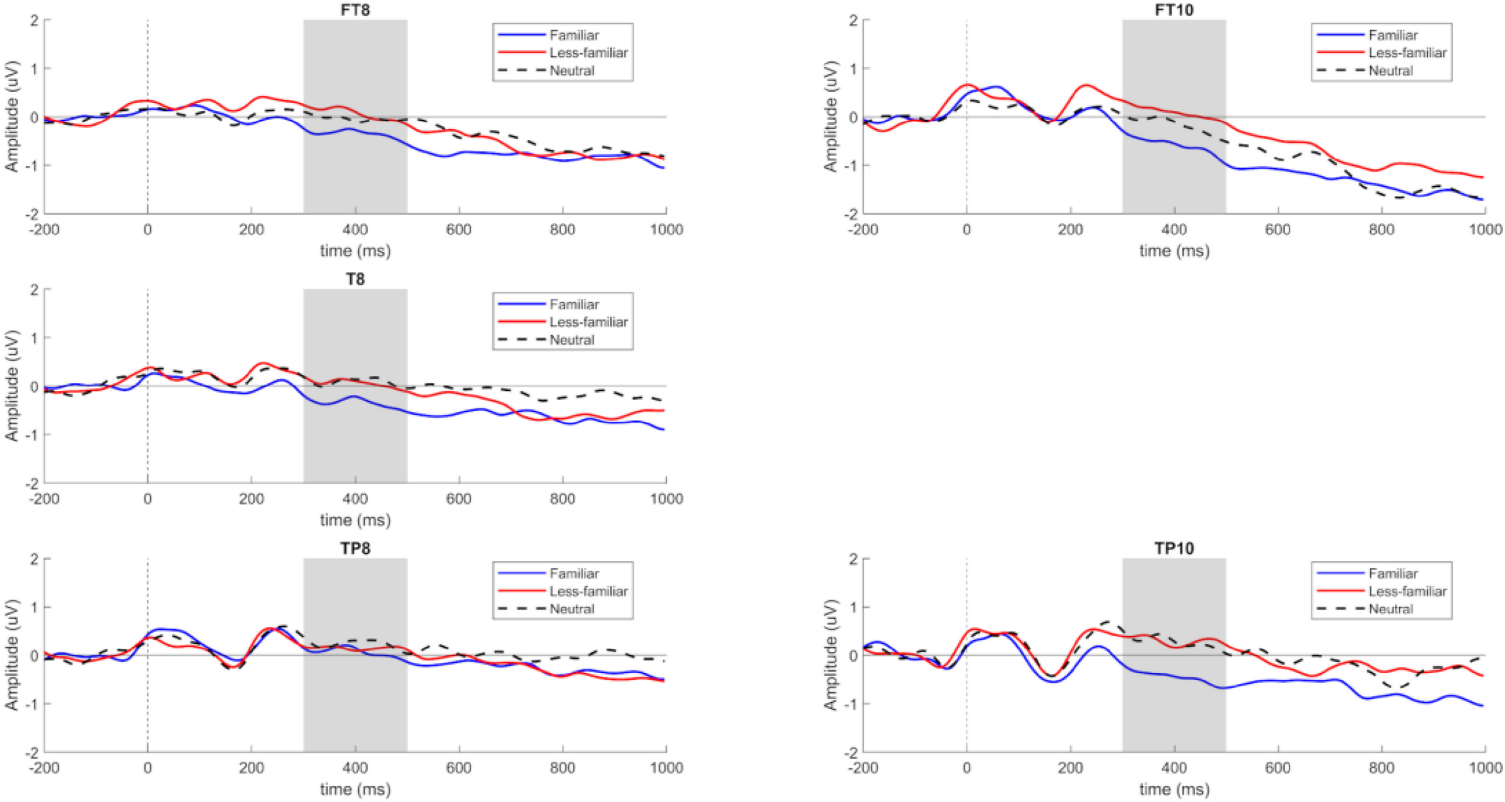
Slow Negativity Potential component of Locus 1 for electrodes FT8–FT10–T8–TP8–TP10 sorted by F, N, and LF conditions (created by the authors).

**FIGURE 3:**
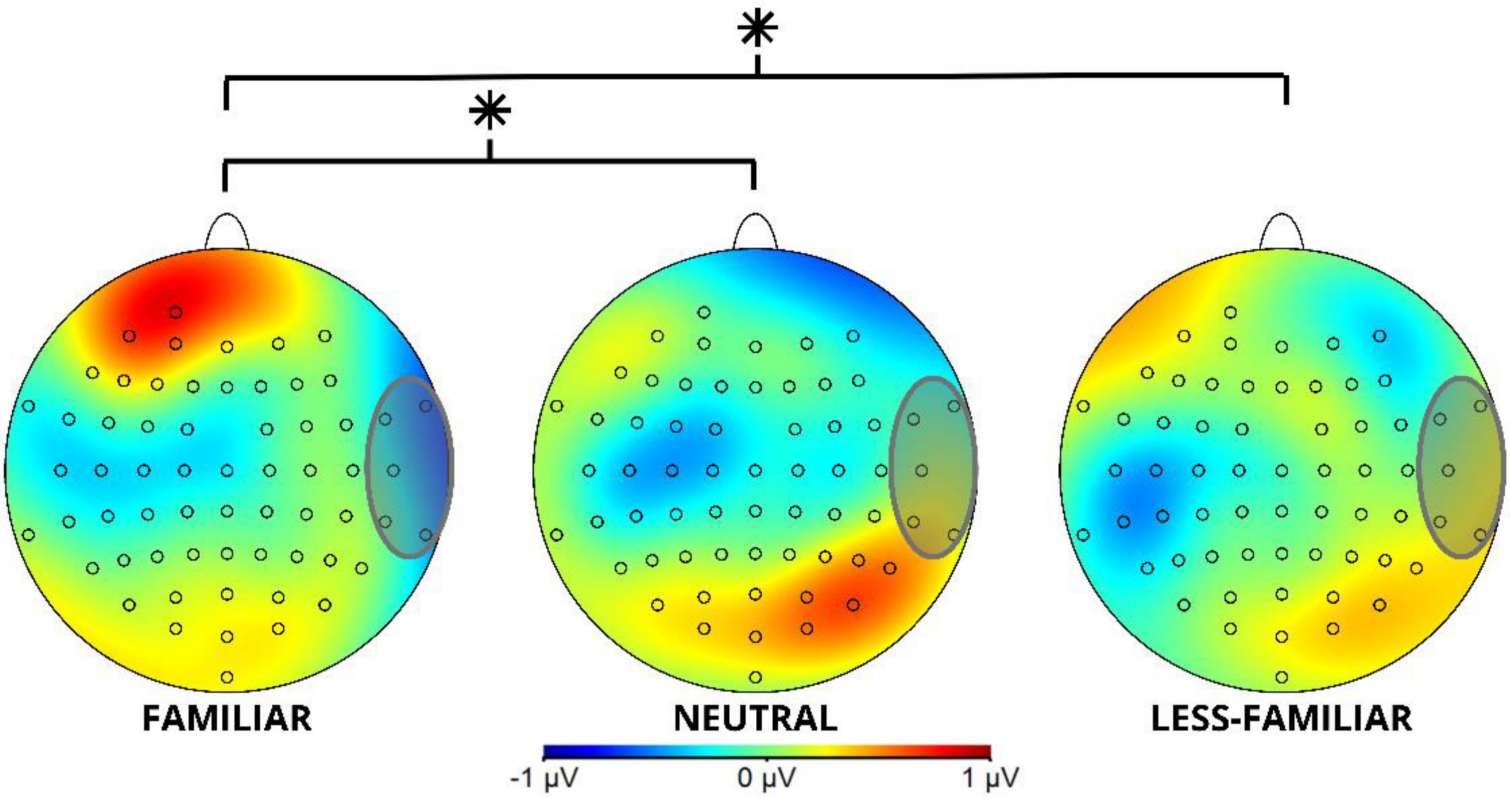
Topographical map of the Slow Negativity Potential component in Locus 1 in the studied 300–500 ms time window for the F, N and LF conditions. The gray circle shows the ROI involving channels FT8, FT10, T8, TP8, and TP10 for the Slow Negativity Potential component. *Indicates a significant difference (*p* < 0.05).

At the critical word 1, we found a slow negativity potential for the ROI comprising electrodes FT8—FT10—T8—TP8—TP10 of the right hemisphere, as shown in Figures 2, 3. Both the right frontal dorsolateral topographical distribution and the late potential in the window where the effect can be seen are evidence of the component.

The analysis of the 300–500 ms time window shows significant differences *F*(2, 114) = 4.368, *p* = 0.015, with a power of 0.876, for the analyzed conditions (F, N, LF; see Table 2).

**TABLE 2:** ANOVA results of mixed—effect model in locus 1: slow negativity potential component.

Variable	Sum Sq	Mean Sq	NumDf	DenDf	*F*-value	*p*-value
Context	7.751	3.875	2	114	4.368	0.015

The contrasts show a significant difference in the comparison between F and LF, *t*(114) = 2.707, *p* = 0.021 with a more negative amplitude for F than for LF. Additionally, a significant difference was found between F and N, *t*(114) = 2.381, *p* = 0.049, where the F context was more negative than N (see Table 3).

**TABLE 3:** Contrast result between contexts in Locus 1.

Contrast	Estimate	SE	df	*t*-value	*p*-value
F—LF	−0.473	0.175	114	−2.707	0.0212
F—N	−0.416	0.175	114	−2.381	0.0492
LF—N	0.057	0.175	114	0.326	0.9432

As shown in Figure 2, the familiar experimental conditions show higher negativity when compared to the less-familiar and neutral conditions.

### 3.2 Locus 2: N400 and post-N400

#### 3.2.1 N400

**FIGURE 4:**
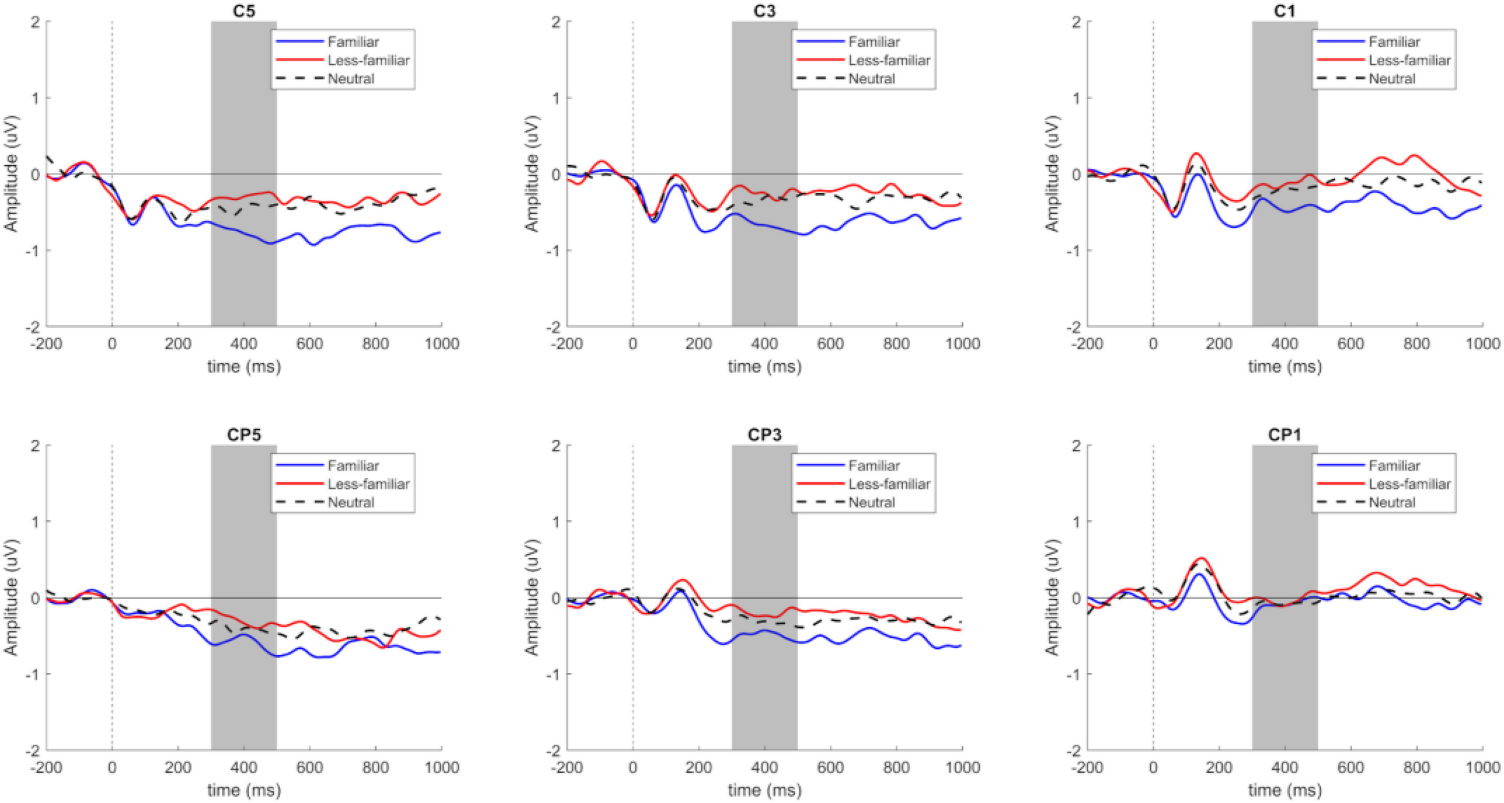
N400 component of locus 2 for electrodes C1–C3–C5–CP1–CP3–CP5, sorted by F, N, and LF conditions (created by the authors).

**FIGURE 5:**
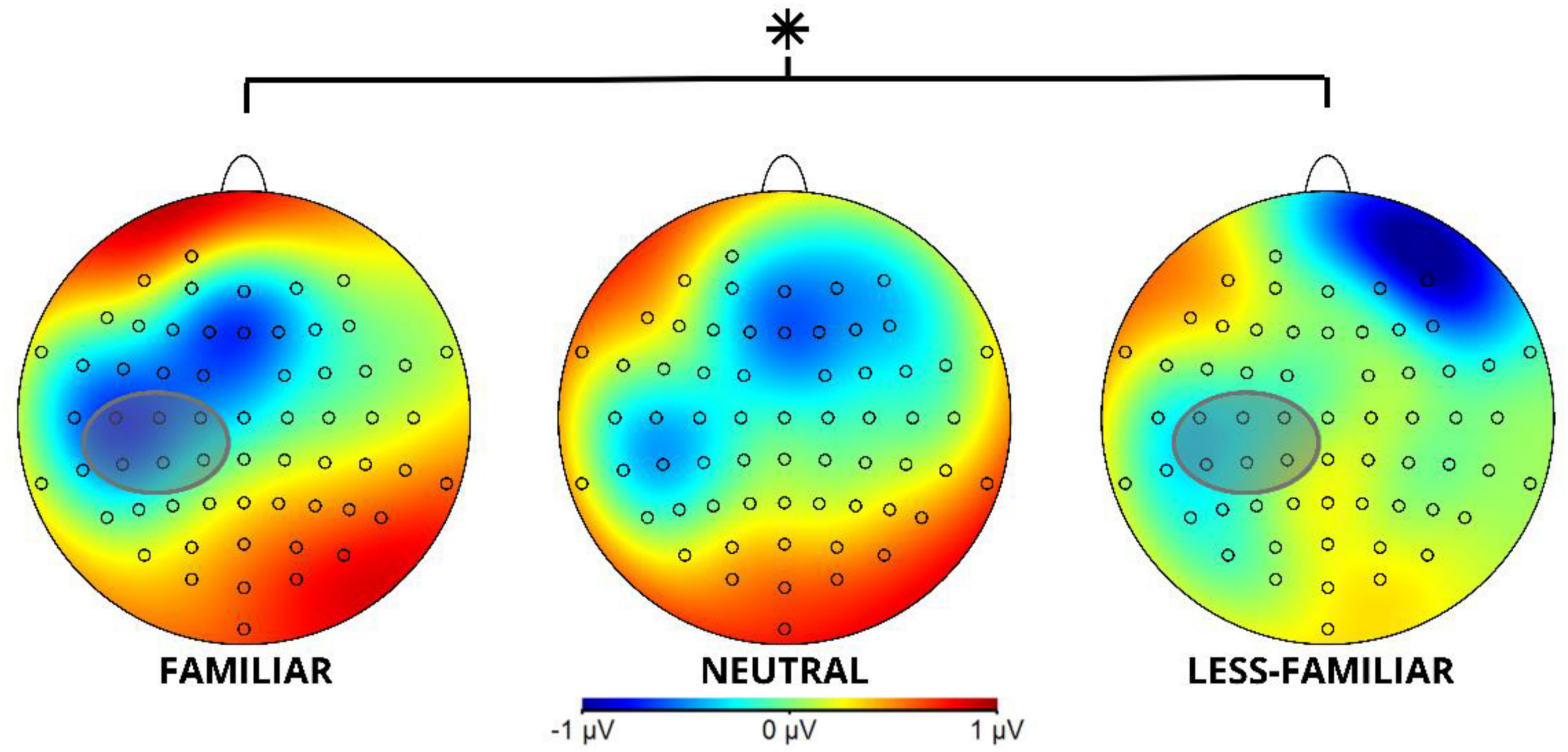
Topographical map of N400 component for locus 2 in the 300–500 ms time window for the F, N and LF conditions. The gray circle shows the ROI involving channels C1, C3, C5, CP1, CP3, and CP5 for N400 component (created by the authors). *Indicates a significant difference (*p* < 0.05).

For critical word 2, an N400 component was detected in the left central parietal region, involving electrodes C1, C3, C5, CP1, CP3, and CP5, as shown in Figure 4. Figure 5 shows the topographical map for each of the conditions.

Based on the ANOVA analyses, we found a significant difference *F*(2, 114) = 3.47, *p* = 0.035, with a power of 0.883 in the analyzed contexts. The contrasts show a significant difference between the conditions F and LF, *t*(114) = −2.59, *p* = 0.029 (see Tables 4, 5).

**TABLE 4:** ANOVA results of mixed – effect model in Locus 2: N400.

Variable	Sum Sq	Mean Sq	NumDf	DenDf	*F*-value	*p*-value
Context	2.779	1.389	2	114	3.468	0.035

**TABLE 5:** Contrast result between contexts in Locus 2: N400.

Contrast	Estimate	SE	df	*t*-value	*p*-value
F—LF	−0.304	0.118	114	−2.590	0.029
F—N	−0.201	0.118	114	−1.710	0.206
LF—N	0.103	0.118	114	0.880	0.654

As shown in Figure 4, there is a higher cognitive cost in the familiar context when compared to the less-familiar context. Likewise, the topographical map shows this negativity with a greater left distribution in the familiar context when compared to the other experimental conditions (Figure 5).

#### 3.2.2 Post-N400

**FIGURE 6:**
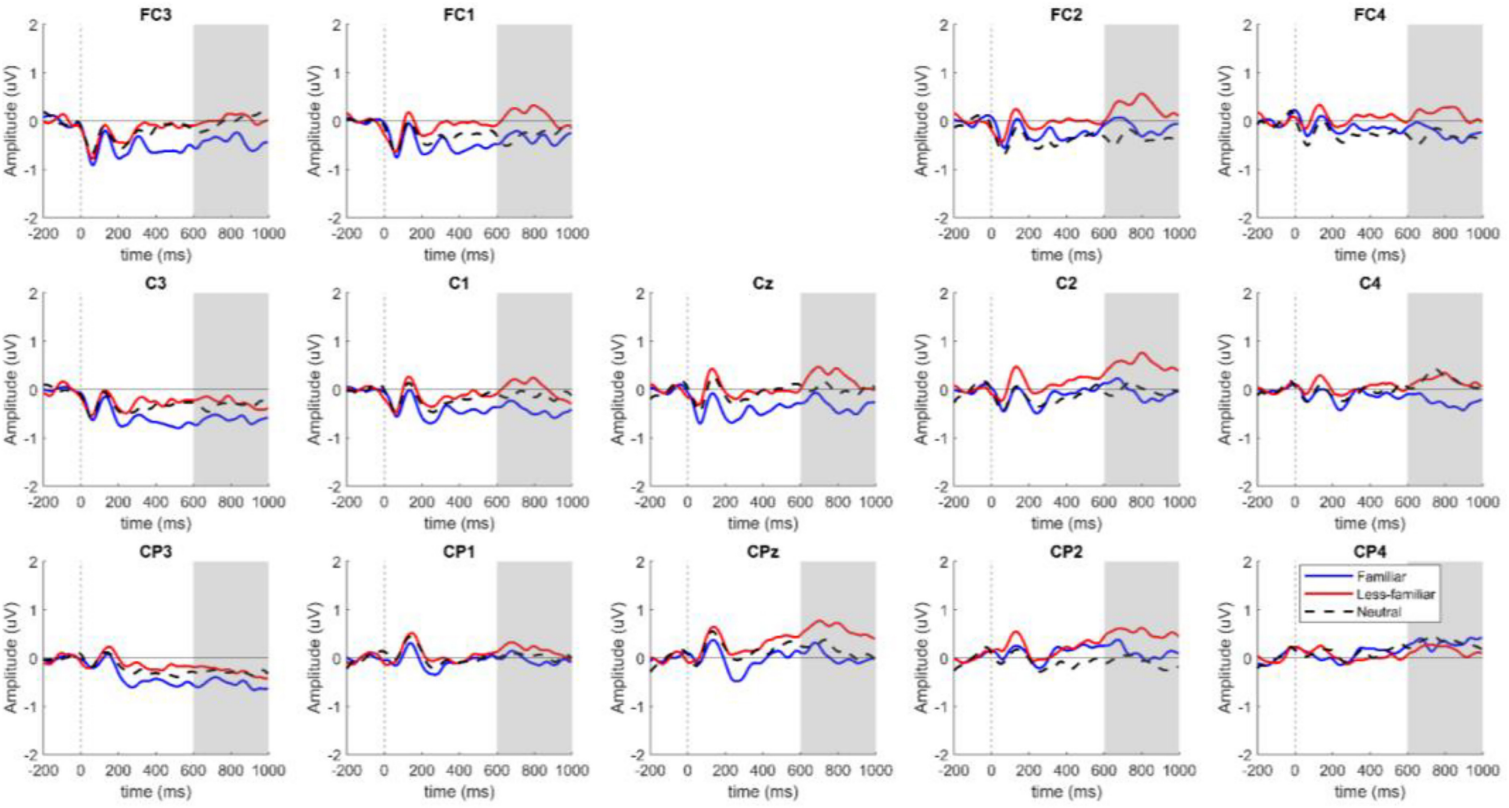
Locus 2 post N400 Component for electrodes FC3, FC1, FC2, FC4, C3, C1, Cz, C2, C4, CP3, CP1, CPz, CP2, CP4 sorted by F, N, and LF conditions (created by the authors).

**FIGURE 7:**
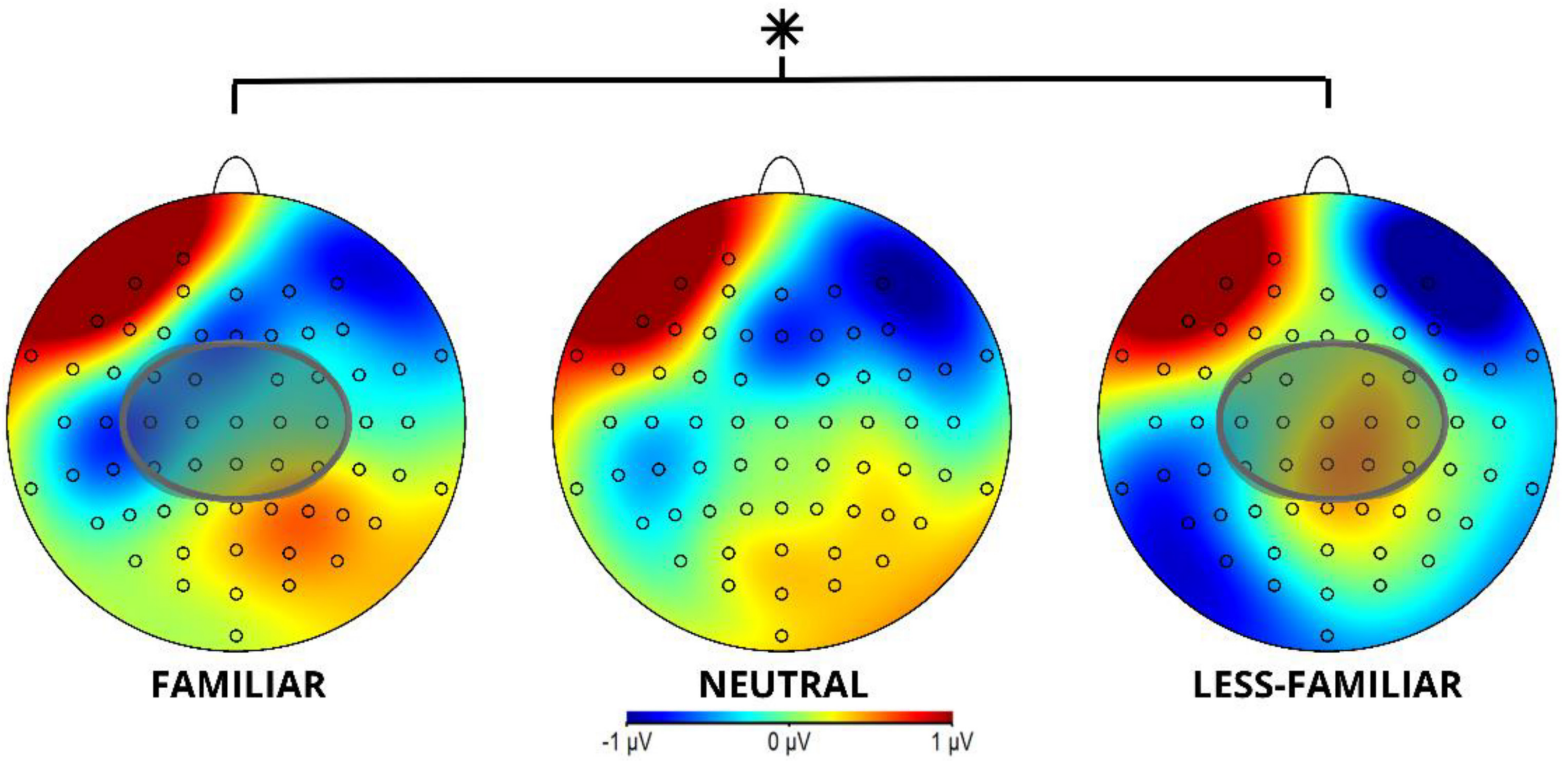
Post N400 component topographical map for locus 2 in the 600-1,000 ms time window for the F, N, and LF conditions. The gray circle shows the ROI involving channels FC3, FC1, FC2, FC4, C3, C1, Cz, C2, C4, CP3, CP1, CPz, CP2, and CP4 for post N400 component (created by the authors). *Indicates a significant difference (*p* < 0.05).

In locus 2 at the 600–1,000 ms time window a post-N400 component was found in the ROI, involving electrodes FC3, FC1, FC2, FC4, C3, C1, Cz, C2, C4, CP3, CP1, CPz, CP2, and CP4, as shown in Figure 6. The analysis of this time window shows a significant difference, *F*(2, 114) = 4.50, *p* = 0.013, with a power of 0.919, for the analyzed conditions (see Table 6 and Figure 7). Contrasts reveal a significant difference between conditions F and LF, *t*(114) = −2.94, *p* = 0.011 (see Table 7).

**TABLE 6:** ANOVA results of mixed—effect model in locus 2: Post N400.

Variable	Sum Sq	Mean Sq	NumDf	DenDF	*F*-value	*p*-value
Context	4.521	2.261	2	114	4.503	0.0131

**TABLE 7:** Contrast result between contexts in Locus 2: Post N400.

Contrast	Estimate	SE	df	*t*-value	*p*-value
F–LF	−0.387	0.132	114	−2.940	0.011
F–N	−0.125	0.132	114	−0.950	0.610
LF–N	0.262	0.132	114	1.990	0.119

### 3.3 Locus 3

#### 3.3.1 Behavioral results: reaction time and accuracy

##### 3.3.1.1 Reaction time

As a main effect, lexicality is statistically significant, *F*(1, 4804.8) = 645.72, *p* < 0.001, i.e., participants took longer to respond to pseudowords than to words, as expected. The context main effect was not significant, *F*(2, 4801.3) = 2.47, *p* = 0.084. As there is a lexicality main effect, we can infer that both variables, word and pseudoword, behave differently. As a result of this, there is no significant context main effect. At a behavioral level, the interaction of the variables context and lexicality was not significant, *F*(2, 4801.3) = 1.02, *p* = 0.36.

Since the interaction was not significant, we explored the results separately by word and pseudoword in order to look at the behavior of each experimental condition. This was done with the same aforementioned method.

**FIGURE 8:**
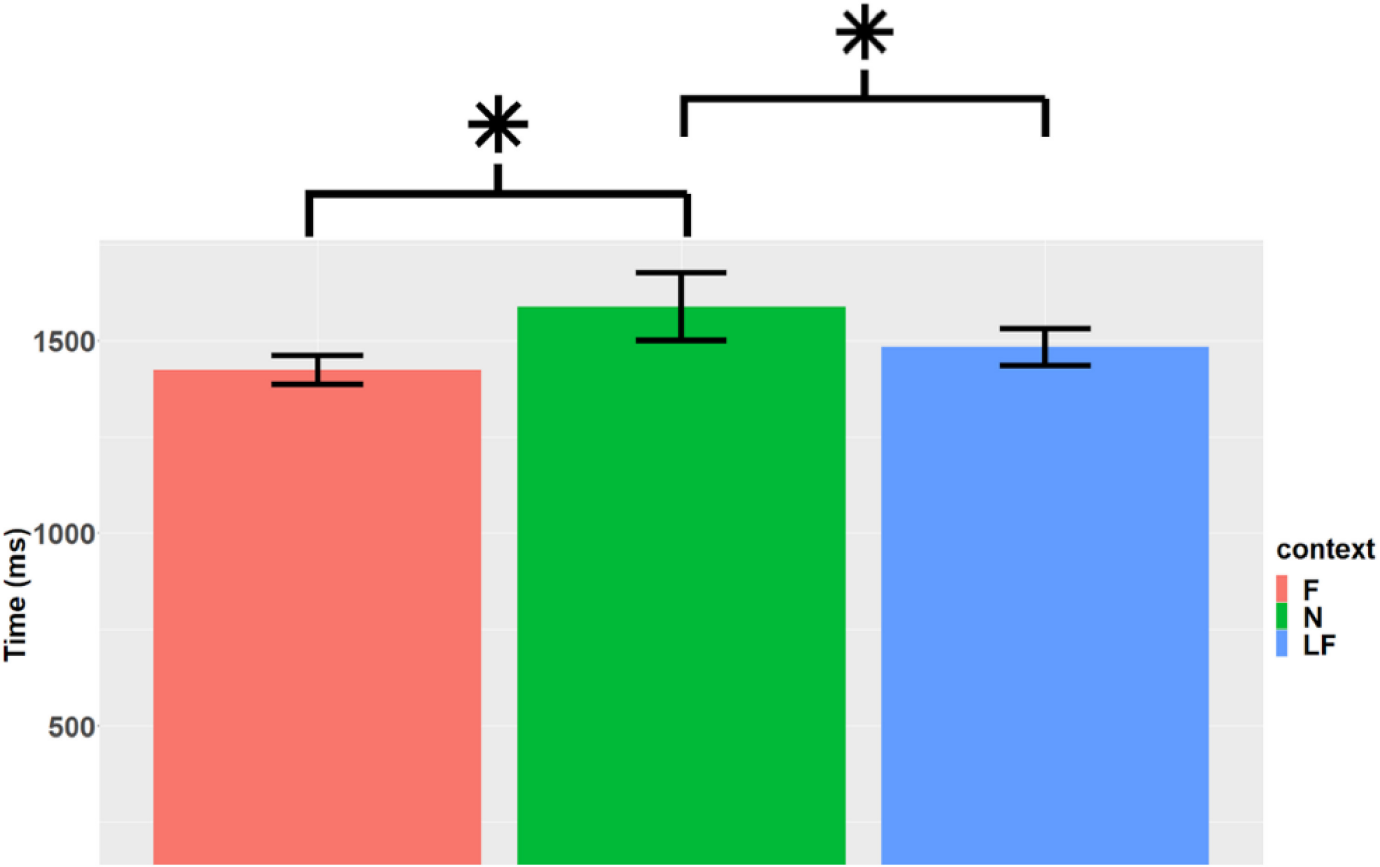
Reaction times according to word context. F(m = 1423.7, SD = 1069.2, se = 36.7), N (m = 1588.7, SD = 2535.3, se = 88.5) and LF (m = 1483.4, SD = 1382, se = 48) (created by the authors). *Indicates a significant difference (*p* < 0.05).

A significant difference was found in relation to the context of the word, *F*(2, 2,360) = 5.23, *p* = 0.005. Contrasts revealed a significant difference between F and N, *t*(2,358) = 3.076, *p* = 0.006, in which students took longer to respond to N than to F. Likewise, there was a significant difference between LF and N, *t*(2,359) = 2.420, *p* = 0.041, in which participants took less time to respond to LF words than to N. Results are displayed in Figure 8. In the case of pseudowords, there is no significant difference associated with context, *F*(2, 2,318) = 0.574, *p* = 0.564.

##### 3.3.1.2 Accuracy

When carrying out a likelihood-ratio test, we found that the model that considers context and lexicality predicts accuracy, X^2^(3) = 30.2, *p* < 0.001. However, no interaction effect can be found between context and lexicality, X^2^(2) = 5.8, *p* = 0.055. Because of this, main effects were analyzed. Additionally, significant effects were found both for context, X^2^(2) = 7.65, *p* = 0.02, and lexicality, X^2^(1) = 22.68, *p* < 0.001.

When considering context in the Tukey method, a significant difference can be found between the F and N contexts. The odds ratio of responding correctly to F targets is higher (OR = 1.97, 95% CI [0.166 1.185]) than to N targets (*p* = 0.02).

Separate lexicality analyses were carried out to look at the differences between words and pseudowords. Regarding context, we found that words are 2.13 times more likely to have a response than pseudowords, *p* < 0.001.

**FIGURE 9:**
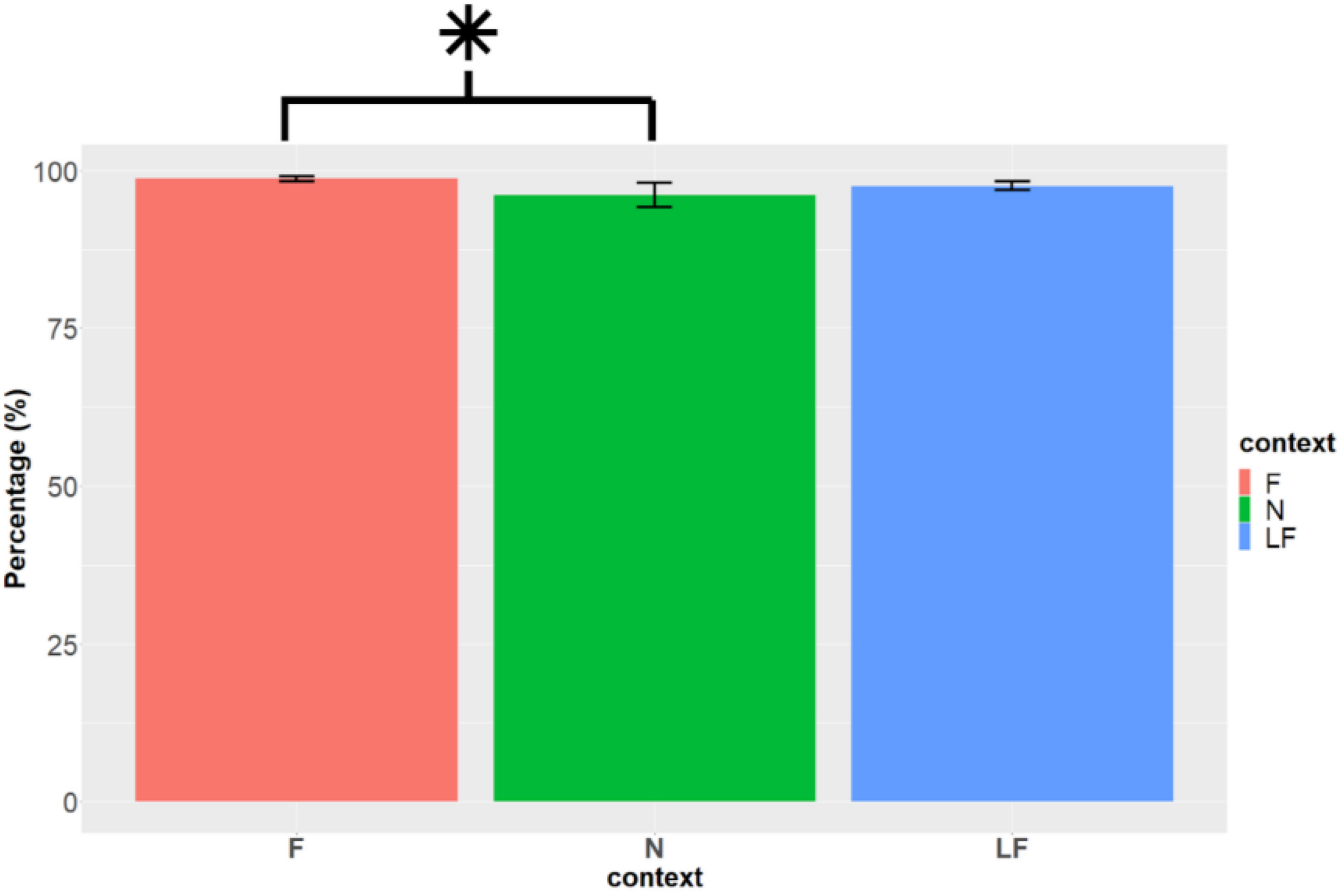
Accuracy (%) according to target word. F(m = 98.7, SD = 3.2, se = 0.42), N (m = 96.2, SD = 14.6, se = 1.93) and LF (m = 97.7, SD = 5.55, se = 0.742) (created by the authors). *Indicates a significant difference (*p* < 0.05).

Since there is a significant main effect, the lexicality factors were analyzed separately. The context effect on word type showed a significant difference X^2^(2) = 13.81, *p* = 0.001. Contrasts revealed a significant difference in the F—N pair, *z* = −3.44, *p* = .00057. This means that the participants were more accurate for F words than for N words, as shown in Figure 9. In the case of pseudowords, there was no significant difference, X^2^(2) = 1.82, *p* = 0.4.

#### 3.3.2 ERP components: FN400 and N400

##### 3.3.2.1 FN400

For locus 3, an FN400 component in the ROI was explored in the 300-500 ms time window, involving electrodes F3, F5, F7, FC3, FC5, FT7. The statistical analysis of the time window showed a significant difference in the context by lexicality interaction, *F*(2, 283.1) = 4.05, *p* = 0.019. In order to analyze the effect of context, the lexicality variables were analyzed separately. However, for word target no significant difference was found by context (F—N—LF), *F*(2, 113.2) = 1.4, *p* = 0.25 (see Table 8).

**TABLE 8:** ANOVA results of mixed—effect model in locus 3: FN400.

Variable	Sum Sq	Mean Sq	NumDf	DenDF	*F-*value	*p-*value
Context	0.486	0.243	2	283.12	0.110	0.896
Lexicality	1.239	1.239	1	283.13	0.559	0.455
Context × lexicality	17.951	8.975	2	283.12	4.047	0.019

##### 3.3.2.2 N400

In the 300–500 ms time window, we explored an N400 component in the ROI involving electrodes C4, C6, CP4, CP6, T8, TP8. Additionally, there is a significant difference in the interaction between the context and lexicality variables, *F*(2, 283.1) = 3.64, *p* = 0.027. However, when analyzed separately, no significant difference was found for word, *F*(2, 113) = 0.32, *p* = 0.73 (see Table 9).

**TABLE 9:** ANOVA results of mixed—effect model in locus 3: N400.

Variable	Sum Sq	Mean Sq	NumDf	DenDF	*F*-value	*p*-value
Context	3.851	1.925	2	283.12	1.848	0.159
Lexicality	1.623	1.623	1	283.13	1.558	0.213
Context × lexicality	7.589	3.795	2	283.12	3.642	0.027

## 4 Discussion

The aim of this study was to explore the costs and benefits of the time course of inferences in university students with reading comprehension difficulties. Our results show the neural time course of inferences at three different loci: (1) a restricted context, marked by familiarity, a less-familiar context and a neutral context; (2) a critical phrase that closes the event proposed within the context; (3) a target word that is inferred from the story being read.

Our hypothesis posed that the context of the stories would influence students from the start of the narration, causing differences between the familiar and less-familiar contexts via an N400 component. Likewise, we expected to see an influence of lexicality in inference generation, leading to a possible FN400 or N400 effect. Below, we discuss each locus based on which neural component was found and its description in literature.

### 4.1 Locus 1: slow negativity potential component

As hypothesized, context had an influence on university students from the start of the narration. In this locus, we found an unexpected electrophysiological component, the slow negativity potential component. While its topographical distribution is mostly frontal central in some research (Delogu et al., 2021; León-Cabrera et al., 2017, 2019), it has also been found in right dorsolateral regions (Frishkoff et al., 2004), as was the case in our study. In line with the proposal of some authors (Calvo, 2000; Nieuwland and Van Berkum, 2006; Wirth et al., 2008), the semantic context had an immediate influence on and marked the course of reading.

Some authors (Frishkoff et al., 2004; Nieuwland and Van Berkum, 2006) have found this potential in two modalities, verbal and auditory, showing the same trend as in our case: higher negativity in a more restricted context, such as familiar contexts, when compared to other contexts. In this study, we found statistically significant contrasts between the familiar and less-familiar contexts, as well as between the familiar and neutral contexts, showing larger negativity in familiar contexts. Apparently, this component is associated with the predictability of upcoming events in stories and can be considered an indicator of semantic prediction in top-down discursive processes (Wirth et al., 2008). It is interesting to note that for the participants of this study, familiar contexts already had a higher prediction cost than other linguistic contexts.

The absence of the N400 component at the first locus, yielding to a sustained Slow Negativity, suggests that this initial stage is primarily associated with the general narrative context. Consequently, situation models linked to familiar contexts are recruited more heavily than the localized semantic processing typically indexed by the N400 (see Pérez et al., 2024). This finding aligns with the notion of impaired resonance processes during the activation of prior knowledge, a hallmark of shallow processing in students with reading comprehension difficulties.

### 4.2 Locus 2: N400 component

An N400 component was found in the typical time window for that neural correlate, between 300 and 500 ms, although it had a left and not a right topographical distribution. Reports of this component in the literature describe it in both hemispheres with an unstable topographical distribution, as its location depends highly on the experimental manipulation (Kutas and Federmeier, 2011; Voss and Federmeier, 2011). This left laterality was also reported by Ding et al. (2016) for emotion verbs in contrast to neutral words and by Holcomb and Mcpherson (1994) in unrelated target images. Likewise, Tabullo et al. (2020) found a left lateral N400 in a narrower time window: 300–400 ms. The authors manipulated the context (strong/weak) and predictability (expected/unexpected) in two university reader groups: skilled readers and readers with reading difficulties. Group inclusion was based on a Cloze test. The results of the study showed higher negativity for unexpected endings, especially when dealing with restricted contexts, in skilled readers. Additionally, they found a higher positivity associated with the post-N400 component in unexpected endings, with a higher prominence in the restricted context, only in skilled readers. These results suggest that readers whose reading skill is high made early predictions, based on a more restricted context that manifested in a reduction of the N400 component for strong contexts with a high predictability. Conversely, readers with reading comprehension difficulties showed less effective prediction mechanisms by not manifesting a reduction of the N400 component and by not eliciting late positivity.

In our experiment, familiar critical phrases elicited higher negativity than less-familiar phrases. Apparently, a familiar context elicited a higher cognitive cost, reflected in the N400 component in the moment that the sentence is being processed. It is important to consider that, unlike in Tabullo et al.’s (2020) experiment, semantic processing was being registered online, as the inference process has not taken place yet, only the effect of the context on the critical phrase. This phrase reveals a higher negativity in familiar contexts as opposed to a less-familiar context. This result might be due to two plausible reasons: an interference cognitive process that takes place in more concrete sentences, represented in a familiarity context, or difficulties to benefit from the context in order to comprehend the story by less skilled readers. We will address the evidence for both of these reasons.

In the case of concreteness of the phrase, Holcomb et al. (1999) found that concrete final words elicited a higher N400 in the 300–500 ms time window and its negativity extended to a later time window, between 500 and 800 ms. The N400 effect in our study could be explained by the fact that familiar words have a larger amount of semantic information than abstract words. Thus, they have a higher cognitive cost. This effect also takes place in the final locus of counterfactual stories with instrumental type events with a double meaning, which implies the use of a specific tool to achieve a goal (Urrutia et al., 2012). In spite of this, the effect of concrete words seems to dissipate in the context with higher expectations (Holcomb et al., 1999), something that does not necessarily happen in our study for the familiar context.

According to Amoruso et al. (2013), expectations depend on online contextual information and the reader’s prior knowledge, whose discrepancies are reduced if both constructs coincide. However, a series of experimental studies (Dalla Volta et al., 2009; De Vega et al., 2013; de Vega and Urrutia, 2011) have reported that in the case of language action, such as what could be found in familiar stories as opposed to less-familiar ones, there is an interference effect when sensorimotor processes take place in parallel to the action. In this case, the familiar stories have a higher sensorimotor representation when compared to less-familiar stories, according to the experience of the reader. This may have caused a higher cognitive cost similar to the interference effect seen in behavioral studies.

Nonetheless, there is a series of studies reporting how readers benefit from context in ERP studies, as previously mentioned in this article (Baggio et al., 2008; Burkhardt, 2007; Ito et al., 2016; Kuperberg et al., 2011). Federmeier et al. (2007) found a graduation of context in three types of contexts: more restrictive, mildly restrictive and unexpected endings. Their results showed an N400 component with a higher negativity in unexpected endings, as opposed to a reduction of the N400 component in more restrictive contexts, when the ending of the sentence adjusted better to the context. Other authors (Wlotko and Federmeier, 2012) went even further by showing a higher graduation of the contextual level for the final word, in a measure that proposed five ranges of likelihood, ranging from 10 to 30%, 30 to 50%, 50 to 75%, 75 to 90%, and 90 to 100%, as opposed to an unexpected ending. Results showed an N400 effect with higher negativity in the unexpected range and a graduation of such negativity according to the five ranges of likelihood, according to the reader’s expectations. Thus, the amplitudes of the N400 component decreased inversely to the increase of restriction.

Previous studies have shown this context graduation taking the opposite direction to that of our study. Because of this, it is worth questioning whether predictive processing associated with context takes place under every circumstance or whether the processes involved in the inhibition and/or review of a prediction in more familiar contexts might be failing in readers with reading comprehension difficulties. It is likely that our students’ processing is rather bottom-up, based on the integration of lexical and semantic information, rather than interactive, where context information is used in advance or “predictively.” This second interpretation of the results will be explained below.

There are few studies that delve into the individual differences in reading comprehension (Tabullo et al., 2020). Individual differences have been found in bilingual groups with higher or lower efficiency, where the latter group showed a greater amplitude of the N400 component and a higher late positivity (LPC component) in sentences with an explicit continuation or paraphrasing, instead of more conceptual variables like inferences (Yang et al., 2018). Another study (Pérez et al., 2015) showed differences between participants with high working memory and those with lower working memory. The results showed that only the former group was able to correct an incorrect contextual interpretation and integrate new information through a higher negativity of the N400, while lower working memory participants faced difficulties to inhibit the initial interpretation and to integrate it into the discourse through a lesser amplitude of the N400 component.

A more specific study, Landi and Perfetti’s (2007), showed how skilled readers benefit from the context in a verbal semantic pairing task, where participants had to choose whether a pair of words was related or not. The results showed a higher negativity in the N400 component for semantically unrelated pairs in both populations. However, the less-skilled readers showed a reduction of N400. Additionally, the polarity of the N400 in this task showed higher negativity in less-skilled readers when compared to skilled readers. This pattern was repeated in pairing tasks involving word-picture pairs and in a phonological task, i.e., deciding whether two words are pronounced in the same manner. This negativity has not been explored in depth. However, it can be seen in our study for the N400 component and the post-N400 component, where polarity associated with the experimental conditions remained more negative in the experimental condition associated with familiarity, displaying the opposite direction to what would be expected. In other words, there was a higher positivity for less-familiar contexts when compared to familiar ones.

The post-N400 component is a reflection of the cost of prediction error that takes place due to a higher inhibition of the preceding words (Cheimariou and Morett, 2023; Van Petten and Luka, 2012). This component arises when there is a conflict between the reader’s expectations and the construction of the situation model in more restrained contexts (Delong et al., 2011; Federmeier et al., 2007; Nieuwland and Van Berkum, 2006; Rodríguez-Gómez et al., 2016; Tabullo et al., 2020). However, the higher positivity in our experiment was found in less-familiar contexts. In this position, we expected to find a P600 component, similarly to the one found in Steele et al. (2013). Nevertheless, the P600 component takes place at an earlier stage, in the 400–800 ms time window, showing a higher deflection at around 600 ms. The post-N400 component has a larger distribution, including frontal, central and parietal regions, which reflect additional processes of semantic integration, such as the resolution of semantic conflicts or re-analyses related to lexical anticipation processes (Van Petten and Luka, 2012). Such cognitive processes seem to be derived from the processing of less-familiar contexts as opposed to familiar ones.

### 4.3 LOCUS 3: lexical decision task

In the third locus of this study, we can truly see when textual inference takes place, since in a lexical decision task readers decide if the following lexical entry is a word or a pseudoword. The word was derived from both contexts, familiar and less-familiar, and did not hold any relation to the neutral context. Pseudowords shared the same phonological structure with the words, as only one syllable was changed and the consonants were replaced by others similar to the ones in the target. Behavioral results of this research reveal no significant interaction effect in reaction times or accuracy, although there was an effect of lexicality leaning toward longer times and lesser accuracy for pseudowords.

By analyzing words separately, just as in Steele et al.’s (2013) study, wherein pseudowords were excluded from the analysis, we found significant differences between the conditions. These differences were evidenced by shorter reaction times for familiar words than for neutral words, and also shorter reaction times for less-familiar words compared to neutral words. Likewise, we found higher accuracy rates for familiar words when compared to neutral ones. These results show that readers benefited from context in order to make an inference. It stands out that in Steele et al.’s (2013) study, words derived from the less-familiar context took less time than words derived from the familiar context, although the difference in contrasts took place between the familiar and the neutral context and between the less-familiar and the neutral context. In our study, we did not find any significant differences between the familiar and the less-familiar context. On the other hand, it is interesting that reaction times of our participants were considerably longer to that of Steele et al.’s (2013) participants. In their results, the authors reported mean reaction times of 570 ms for the familiar condition, as opposed to 1,423 ms for the familiar condition reported in our experiment. This was also the case for the other variables in which less-skilled readers virtually took twice as long.

In the electrophysiological analysis, we found a statistically significant interaction in the FN400 component. However, in the separate analysis, we found no significant differences in the word variable. As mentioned above, the FN400 component is a neural correlate of familiarity (Bader et al., 2023; Bridger et al., 2012; Leynes and Upadhyay, 2022; Shayesteh et al., 2020; Stróżak et al., 2021). Due to this, we expected to find some effect on this component. Nevertheless, lexicality seems to interact with context and less-skilled readers paid special attention to pseudowords. These effects can be explained by Perfetti (1985, 1994) theory, wherein lexical access in less-skilled readers is very slow, as reflected by their reaction times. This causes said readers to have enough time for contextual information to start to flow on lexical access.

In regard to the N400 component, statistically significant effects were once again found for the context x lexicality interaction, although the effects occurred only for pseudowords. By contrast, in Steele et al.’s (2013) study, there was a higher negativity in neutral contexts compared to the familiar and less-familiar experimental conditions for words. Finally, the authors found the P600 component in this position, revealing a reinterpretation effect for familiar and less-familiar contexts in relation to neutral contexts. However, in our case, there was no late effect in the third locus.

An important difference between Steele et al.’s (2013) study and our study is in the SOA interval between the final phrase and the target word, since the researchers were looking for predictive inferences in a 1,000 ms inter-stimulus timeframe. Conversely, in our study, we were looking for automatic inferences, without inter-stimulus interval, in order to record bridging or forward inferences establishing causal coherence. Moreover, it is worth noting that the sample of this study was made up of less-skilled readers, who have difficulties to make inferences connecting distant contents in a text (Domínguez et al., 1999).

On the other hand, the restrictions of the familiar context in a self-administered task, due to the process of reading and the presentation time of the target word, favor the generation of online inferences (Graesser et al., 1994; Wlotko and Federmeier, 2015). Support for this notion can be found in McKoon and Ratcliff (1992, 1995) who posit that such inferences are generated if information is readily available from general knowledge, as is the case in familiar contexts. The familiarity effects occur for words in the behavioral task and are different from the original study (Steele et al., 2013), where the less-familiar context saw higher facilitation in reaction times for the target word, which took place within time limits, as opposed to our results.

The absence of the P600 component in less-skilled readers can be interpreted through the Validation stage of the RI-Val model. While resonance and integration processes are triggered, evidenced by the context effects in earlier loci, these readers appear to possess a lower coherence threshold. This leads to a failure in the validation phase, where semantic conflicts are not actively monitored or resolved. Furthermore, as suggested by Xu et al. (2023), the lack of a significant P600 effect may indicate that original predictive inferences remain active in the reader’s memory representation, even when they are not confirmed by the text. Consequently, the lack of a P600 suggests that the strategic re-analysis necessary for top-down monitoring is bypassed in favor of a shallow, bottom-up integration that prioritizes local lexical features over global narrative consistency both in locus 2 and in locus 3. In locus 1, however, readers seem to benefit from context.

According to current theories on discourse comprehension, reading processes are partially incremental, flexible and dependent on context (Kutas and Federmeier, 2011). For this reason, there is a high likelihood that, based on the context declared in locus 1 of this research, some semantic content may have generated an expectation from a word. In turn, this may have generated a conflict in the following locus through the N400 and post-N400 components (Altmann and Kamide, 1999). As a result, a combination of both types of processing—top-down in locus 1 and locus 3 in the behavioral task, and bottom-up in locus 2 and 3 in electrophysiology—may coexist in the case of less-skilled readers, for whom the high cost of integrating the current word into the situation model monopolizes cognitive resources. This creates a processing bottleneck where the system is forced into a necessity-driven bottom-up mode. Consequently, these readers remain constrained by the demands of lexical decoding and integration—as indexed by the N400—precluding their ability to utilize contextual information predictively for subsequent discourse. Good readers, on the other hand, use preferentially top-down processing, benefiting from semantic networks in the use of context, as well as bottom-up processing (Tabullo et al., 2020).

## 5 Conclusion

Ultimately, the time course of inferences in less-skilled readers shows the benefits of context through a use of a slow negativity potential component in the first locus, which is associated with higher negativity in familiar contexts. Conversely, in the second locus we see the cognitive cost that familiar context entails, eliciting a higher negativity in the N400 and post-N400 component. The latter component has been associated with lexical anticipation processes rather than contextual anticipation processes of discourse (Van Petten and Luka, 2012). Lastly, in the third locus, participants benefited from context in the behavioral task by generating automatic inferences associated with a higher facilitation of words from familiar and less-familiar contexts, as opposed to a neutral one. Nonetheless, no effect can be seen in the electrophysiological components at word level, only in pseudowords, showing the benefit of context for such lexical entries, as well as the cost of lexicality in the semantic processing of words.

One of the limitations of this study is not having a group of skilled readers to establish a direct contrast between both populations. This implies that all interpretations of this study must be taken with care, as no comparisons can be made with the current sample. An additional limitation is the lack of SOA graduation studies, i.e., two experiments with the same population comparing a short SOA with a long SOA in order to establish a clearer difference between automatic and predictive inferences. In future research, the combination of electrophysiological techniques—used mainly in contextual semantic processing—with eye movement technique, allowing for a better monitoring of lexical and even sublexical processes, might shed more light on the costs and benefits of experimenting with less-skilled readers, as opposed to skilled readers.

## Data Availability

The datasets presented in this article are not readily available because of ethical restrictions. Requests to access the datasets should be directed to maurrutia@udec.cl.
